# Age related gene DST represents an independent prognostic factor for MYCN non-amplified neuroblastoma

**DOI:** 10.1186/s12887-021-02753-6

**Published:** 2021-06-11

**Authors:** Haiwei Wang, Xinrui Wang, Liangpu Xu, Ji Zhang, Hua Cao

**Affiliations:** 1 Medical Research Center, Fujian Maternity and Child Health Hospital, Affiliated Hospital of Fujian Medical University, Fuzhou, Fujian China; 2 State Key Laboratory for Medical Genomics, Shanghai Institute of Hematology, Rui-Jin Hospital Affiliated to School of Medicine, Shanghai Jiao Tong University, Shanghai, China

**Keywords:** Pediatric neuroblastoma, MYCN amplification, Age, DST, TARGET, GEO

## Abstract

**Background:**

MYCN amplification and age are two critical prognostic factors of pediatric neuroblastoma. Previously, we had revealed the prognosis of MYCN target genes. However, the prognostic effects of age related genes in neuroblastoma are unclear.

**Methods:**

The prognostic significance of age and MYCN amplification was determined through multivariate cox regression and Kaplan-Meier survival analysis. Genes differentially expressed in MYCN non-amplified younger neuroblastoma patients were identified using Therapeutically Applicable Research to Generate Effective Treatments (TARGET) and Gene Expression Omnibus (GEO) datasets. The prognostic effects of age related genes ALCAM, CACNA2D3, DST, EPB41L4A and KIF1B in pediatric neuroblastoma patients were determined by Kaplan-Meier survival.

**Results:**

In a pediatric pan-cancer analysis**,** age was associated with the overall survival of pediatric B-lineage acute lymphoblastic leukemia, neuroblastoma and wilms tumor in TARGET dataset. Moreover, the prognostic effects of age in neuroblastoma were validated using two independent neuroblastoma cohorts. Furthermore, age and MYCN amplification were independent prognostic factors in pediatric neuroblastoma. Compared with MYCN non-amplified older neuroblastoma patients, MYCN non-amplified younger neuroblastoma patients had better clinical outcomes. ALCAM, CACNA2D3, DST, EPB41L4A and KIF1B were highly expressed in MYCN non-amplified younger neuroblastoma patients. And the higher expression levels of ALCAM, CACNA2D3, DST, EPB41L4A or KIF1B were associated with better prognosis of MYCN non-amplified neuroblastoma patients. DST was an independent prognostic factor in MYCN non-amplified neuroblastoma patients and MYCN non-amplified neuroblastoma younger patients with higher DST expression levels had the best clinical overall survival.

**Conclusions:**

Age related gene DST was an independent prognostic factor in MYCN non-amplified neuroblastoma. MYCN non-amplified younger neuroblastoma patients with higher DST expression levels had the best clinical overall survival.

## Background

Pediatric neuroblastoma is a heterogeneous disease with distinctive clinical outcomes [1, 2]. MYCN amplification is an important prognostic factor in neuroblastoma [3]. Pediatric neuroblastoma patients with MYCN amplification are associated with poor prognosis [4–6]. MYCN belongs to the MYC transcription factor family and regulates the expression levels of multiple target genes [7]. Independent of MYCN amplification, MYCN expression levels [8] and MYCN regulated signature [9] are also correlated with the unfavorable outcomes of neuroblastoma. Using published gene expression datasets, previously, we had identified six MYCN target genes which could be used to predict the clinical outcomes of neuroblastoma [10]. Moreover, MYCN amplification represents a therapeutic target in neuroblastoma. Bromodomain inhibitors have specific efficacy in MYCN amplified neuroblastoma [11–13].

Besides MYCN amplification, age at initial neuroblastoma diagnosis is also a prognostic factor [14]. Compared with younger neuroblastoma patients, older neuroblastoma patients usually had worse prognostic outcomes [15]. However, the relationships of age and MYCN amplification in the predication of the clinical outcomes of neuroblastoma are unclear. Also, the transcriptional profiling associated with age and MYCN amplification is unknown.

Here, using TARGET [16], GSE49710 [17] and GSE85047 datasets [18], the transcriptional profiling of MYCN non-amplified younger neuroblastoma patients was identified. Also, the prognostic effects of age and MYCN amplification related genes in neuroblastoma were determined. Our analysis revealed that MYCN non-amplified neuroblastoma was heterogeneous and could be divided into sub-groups based on the age at initial neuroblastoma diagnosis or the expression levels of DST. MYCN non-amplified younger patients with higher DST expression levels had the best clinical overall survival in neuroblastoma.

## Methods

### Data collection

The TARGET datasets were downloaded from https://ocg.cancer.gov/. The GSE49710 and GSE85047 datasets were downloaded from https://www.ncbi.nlm.nih.gov/geo/. All the data was further processed using R software (version 3.5.0; https://www.r-project.org/).

### Prognostic effects of age and MYCN amplification

Neuroblastoma patients were divided into older and younger groups based on the mean age at initial diagnosis. The mean age of neuroblastoma patients at initial diagnosis in TARGET, GSE49710 or GSE85047 dataset was 2.5 years old, 2.1 years old or 2.2 years old, respectively. Kaplan-Meier estimator in GraphPad Prism software (version 5.0; https://www.graphpad.com/) was applied to determine the different clinical overall survival of neuroblastoma patients with different age or MYCN amplification status using TARGET, GSE49710 and GSE85047 datasets. *P* values were determined by Log-rank test.

### Heatmap

The differentially expressed genes in MYCN non-amplified younger neuroblastoma patients were presented using heatmaps by R software “pheatmap” package (version 1.0.12; https://cran.r-project.org/web/packages/pheatmap/ index.html).

### Venn diagram

The common genes which were differentially expressed in MYCN non-amplified younger neuroblastoma in TARGET, GSE49710 and GSE85047 datasets were determined by VENNY 2.1 software (http://bioinfogp.cnb.csic.es/tools/venny/index.html).

### Kyoto encyclopedia of gens and genomes (KEGG) signaling pathway and transcription factors enrichment analysis

MYCN non-amplified younger neuroblastoma associated KEGG signaling pathways [19–21] and transcription factors were determined using The Database for Annotation, Visualization and Integrated Discovery (DAVID) website (version 6.8; https://david.ncifcrf.gov) [22, 23].

### Prognostic effects of ALCAM, CACNA2D3, DST, EPB41L4A and KIF1B

Neuroblastoma patients were divided into two sub-groups based on the mean expression levels of ALCAM, CACNA2D3, DST, EPB41L4A or KIF1B. R software ‘survival’ package (version 3.1–8; https://cran.r-project.org/web/packages/survival/ index.html) was used to test the clinical overall survival of neuroblastoma with higher expression levels or lower expression levels of ALCAM, CACNA2D3, DST, EPB41L4A and KIF1B. Log-rank test was used to determine the *P* values.

### Univariate and multivariate cox regression

The prognosis of age in different pediatric tumor types was determined by univariate cox regression analysis. The associations of ALCAM, CACNA2D3, DST, EPB41L4A and KIF1B in MYCN non-amplified neuroblastoma patients were tested by multivariate cox regression analysis using R software ‘survival’ package ‘coxph’ method (version 3.1–8).

### Correlation plot

R software ‘corrplot’ package (version 0.84; https://cran.r-project.org/web/packages/corrplot/index.html) was used to determine the correlation of ALCAM, CACNA2D3, DST, EPB41L4A and KIF1B based on their expression levels in TARGET, GSE49710 and GSE85047 datasets.

### Statistical analysis

The box plots were generated from GraphPad Prism software. Statistical analysis was performed using the two tails paired Student’s t test in R software. *P* value less than 0.05 was chosen to be statistically significant difference.

## Results

### Age is associated with the overall survival of pediatric neuroblastoma patients

**Fig. 1:**
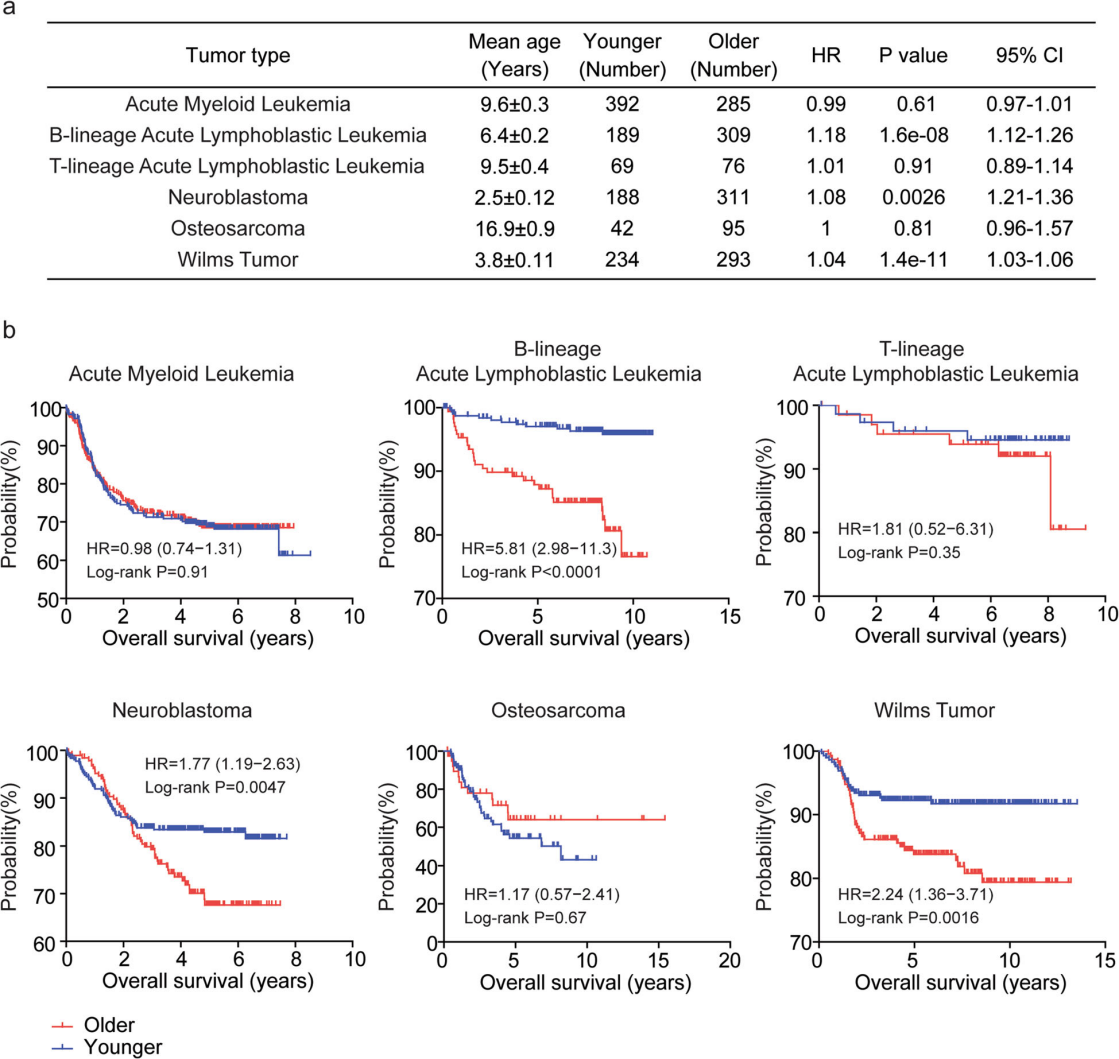
Age is associated with the overall survival of pediatric neuroblastoma patients. **a** Univariate cox regression was used to reveal the prognostic significance of age in different pediatric tumor types in TARGET dataset. **b** The Kaplan-Meier curves showed the different overall survival of older (red) and younger (blue) tumor patients in each pediatric tumor type using TARGET dataset. *P* value was determined by Log-rank test. HR represented the hazard ratio

Previously, we revealed the prognostic effects of age in each adult tumor type using The Cancer Genome Atlas (TCGA) datasets [24]. Here, using TARGET datasets generated from St Jude Children’s Research Hospital, the prognostic effects of age in each pediatric tumor type were further analyzed. Collectively, 2419 patients from six pediatric tumor types were used for further studies. Although, those six tumor types were all developed in children or adolescents, the mean age was quite different (Fig. 1a). The mean age of neuroblastoma patients was 2.5 years old, much younger than other patients. And osteosarcoma represented the most old pediatric cancer type (Fig. 1a).

First, using univariate cox regression analysis, we found that age was significantly associated with the overall survival in pediatric B-lineage acute lymphoblastic leukemia, neuroblastoma and wilms tumor patients (Fig. 1a). However, age had no prognostic effect in pediatric acute myeloid leukemia, T-lineage acute lymphoblastic leukemia or osteosarcoma patients (Fig. 1a).

Similar results were obtained through the Kaplan-Meier survival analysis that age was a critical prognostic factor for pediatric B-lineage acute lymphoblastic leukemia, neuroblastoma and wilms tumor (Fig. 1b). Particularly, age was most associated with the overall survival of pediatric B-lineage acute lymphoblastic leukemia patients (*P* < 0.0001 and HR = 5.81) (Fig. 1b). However, the overall survival of older acute myeloid leukemia, T-lineage acute lymphoblastic leukemia or osteosarcoma patients was not significantly different from younger patients (Fig. 1b).

### Age and MYCN amplification are independent prognostic factors in pediatric neuroblastoma

**Fig. 2:**
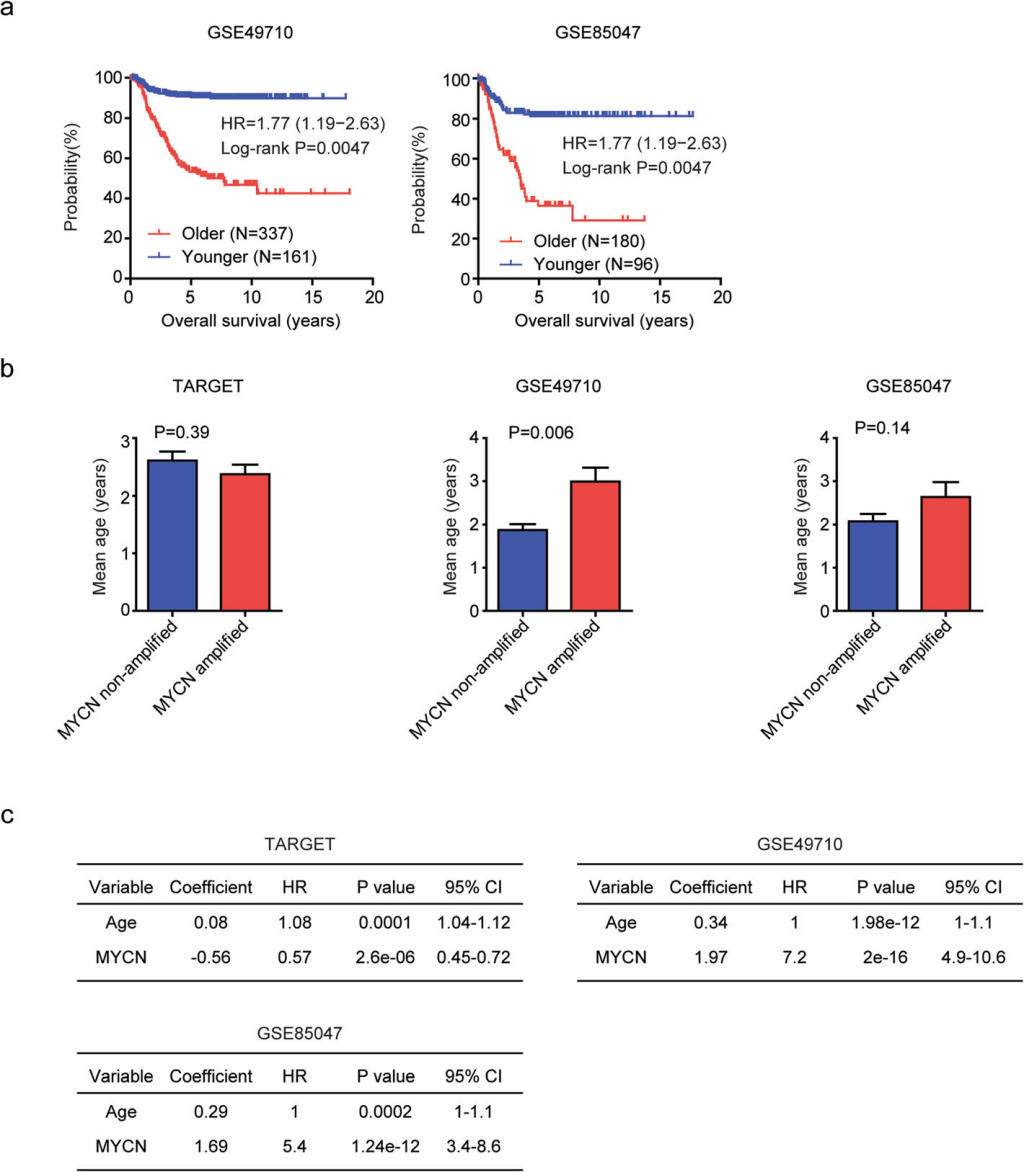
Age and MYCN amplification are independent prognostic factors in pediatric neuroblastoma. **a** Overall survival was analyzed in older (red) and younger (blue) pediatric neuroblastoma patients in GSE49710 and GSE85047 datasets. *P* values were generated from Log-rank test. **b** Box plot showed the mean age of MYCN amplified (red) and MYCN non-amplified (blue) pediatric neuroblastoma patients. P values were determined using the Student’s t test. **c** Multivariate cox regression was used to test the prognostic relevance of age and MYCN amplification in neuroblastoma, using TARGET, GSE49710 and GSE85047 datasets

The prognostic effects of age in pediatric neuroblastoma were further confirmed using 498 Chinese primary neuroblastomas deposited in GSE49710 dataset and 283 primary neuroblastomas deposited in GSE85047 dataset assembled by the European Neuroblastoma Research Consortium (NRC). The mean age of neuroblastoma patients in GSE49710 and GSE85047 datasets was 2.1 and 2.2 years old, respectively. Similar to the results derived from TARGET dataset, we found that, compared with younger pediatric neuroblastoma, older pediatric neuroblastoma patients had worse clinical outcomes in both GSE49710 and GSE85047 datasets (Fig. 2a).

MYCN amplification was also associated with the clinical outcomes of pediatric neuroblastoma [5]. So, next, we determined the relationships of age and MYCN amplification in neuroblastoma. We found that there was no significant difference of the mean age in pediatric neuroblastoma patients with or without MYCN amplification in TARGET and GSE85047 datasets (Fig. 2b). However, compared with neuroblastoma patients without MYCN amplification, the mean age in MYCN amplified neuroblastoma patients was higher in GSE49710 dataset (Fig. 2b). Furthermore, the multivariate cox regression assay suggested that age and MYCN amplification were independent prognostic factors in pediatric neuroblastoma in TARGET, GSE49710 and GSE85047 datasets (Fig. 2c).

### MYCN non-amplified younger neuroblastoma patients have better prognosis

**Fig. 3:**
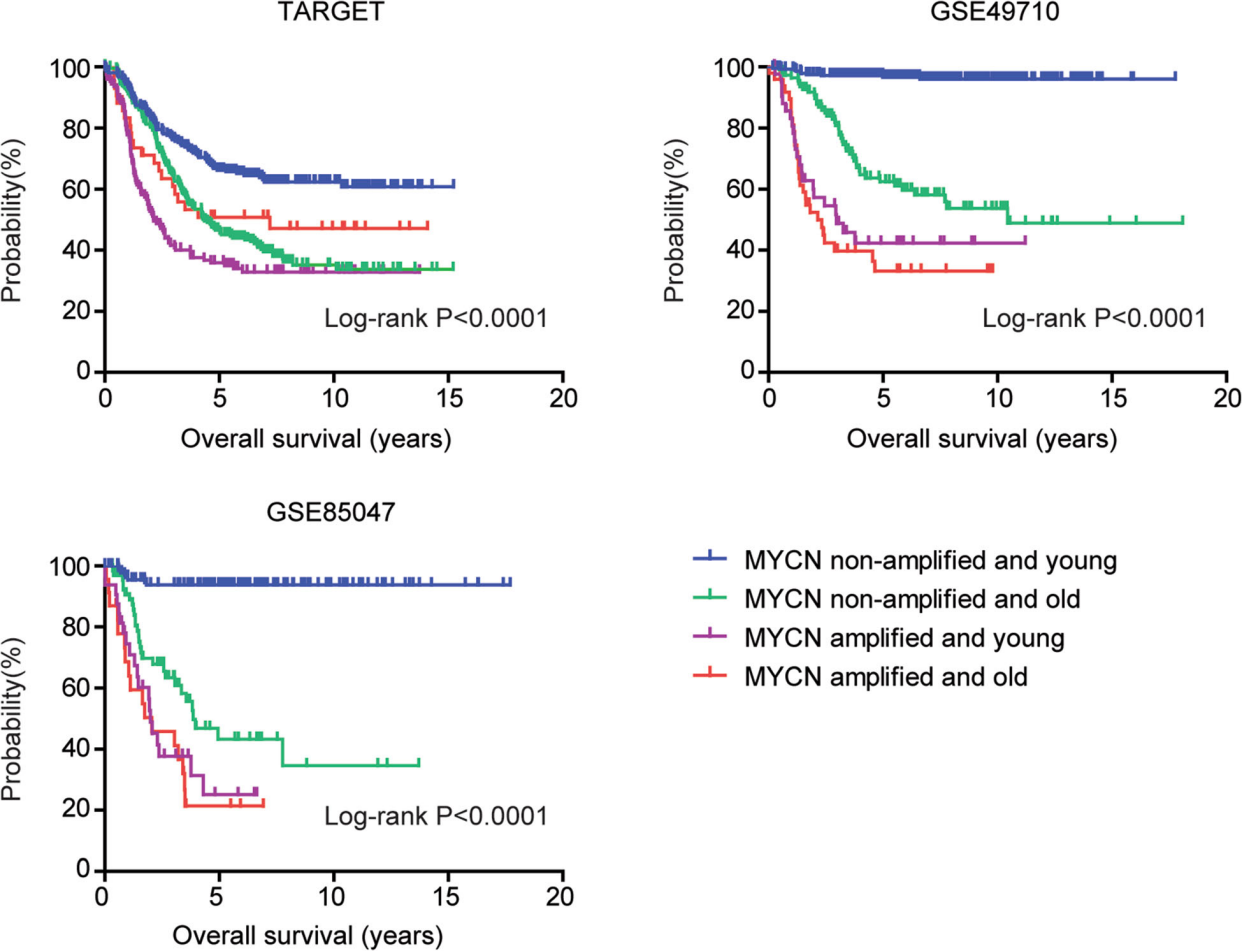
MYCN non-amplified younger neuroblastoma patients have better prognosis. Pediatric neuroblastoma patients in TARGET, GSE49710 and GSE85047 datasets were divided into MYCN amplified older, MYCN amplified younger, MYCN non-amplified older and MYCN non-amplified younger four sub-groups. The Kaplan-Meier plots showed the different overall survival of those four sub-groups. P values were determined using Log-rank test. HR represented the hazard ratio

Next, we determined the combination of age and MYCN amplification in the predication of the clinical outcomes of pediatric neuroblastoma. Pediatric neuroblastoma patients in TARGET, GSE49710 and GSE85047 datasets were divided into four sub-groups based on the mean age and MYCN amplification status. The Kaplan-Meier plots showed that MYCN amplified older patients and MYCN amplified younger patients had not significantly different clinical outcomes (Fig. 3). However, MYCN non-amplified younger neuroblastoma patients had significantly favorable prognosis than MYCN non-amplified older neuroblastoma patients in TARGET, GSE49710 and GSE85047 datasets (Fig. 3). Those results suggested that MYCN non-amplified pediatric neuroblastoma was heterogeneous, and could be divided into older and younger pediatric neuroblastoma sub-groups.

### Identification of the differentially expressed genes in MYCN non-amplified younger neuroblastoma patients

**Fig. 4:**
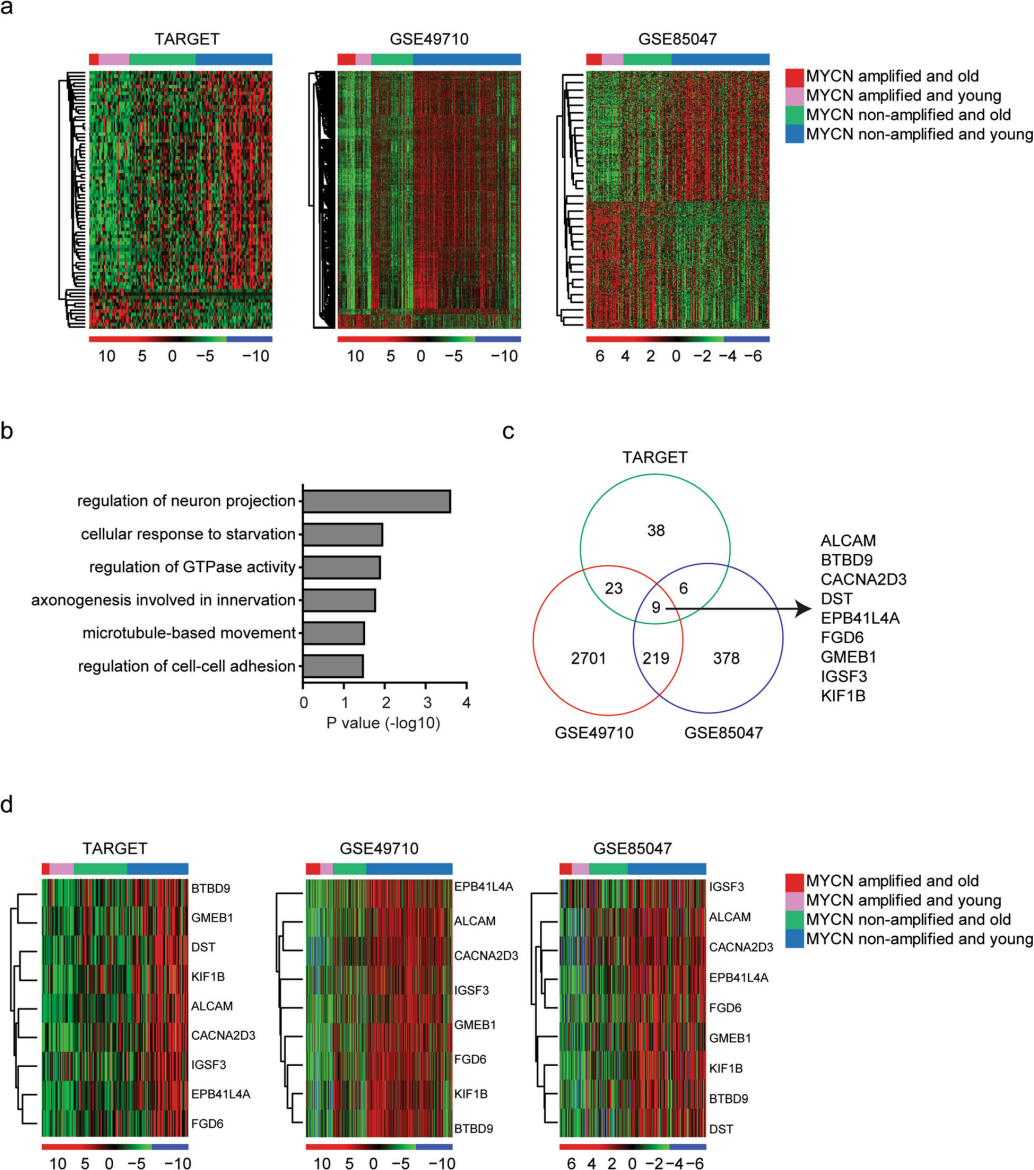
Identification of the differentially expressed genes in MYCN non-amplified younger neuroblastoma patients. **a** Heatmaps demonstrated the differentially expressed genes in MYCN non-amplified younger neuroblastoma patients in TARGET, GSE49710 and GSE85047 datasets. **b** The enriched signaling pathways from the differentially expressed genes in MYCN non-amplified younger neuroblastoma patients in TARGET dataset. **c** Venn diagram depicted the common genes which were differentially expressed in MYCN non-amplified younger neuroblastoma patients in TARGET, GSE49710 and GSE85047 datasets. **d** Heatmaps demonstrated the expression levels of the common genes in TARGET, GSE49710 and GSE85047 datasets

Moreover, we tried to identify the differentially expressed genes in MYCN non-amplified younger neuroblastoma patients in TARGET dataset. Compared with MYCN amplified neuroblastoma patients and MYCN non-amplified older neuroblastoma patients, 64 genes were highly expressed in MYCN non-amplified younger neuroblastoma patients in TARGET dataset (Fig. 4a). However, there were only 12 genes were down-regulated in MYCN non-amplified younger neuroblastoma patients (Fig. 4a). Those differentially expressed genes were involved in the regulation of neuron projection, the regulation of GTPase activity and the regulation of cell-cell adhesion (Fig. 4b).

The differentially expressed genes in MYCN non-amplified younger neuroblastoma patients in GSE49710 and GSE85047 datasets were also identified. There were 2952 and 612 genes were differentially expressed in MYCN non-amplified younger neuroblastoma patients in GSE49710 and GSE85047 datasets, respectively (Fig. 4a). Among them, ALCAM, BTBD9, CACNA2D3, DST, EPB41L4A, FGD6, GMEB1, IGSF3 and KIF1B were commonly changed in MYCN non-amplified younger neuroblastoma patients in TARGET, GSE49710 and GSE85047 datasets (Fig. 4c). Using heatmaps, we showed that those genes were all over-expressed in MYCN non-amplified younger neuroblastoma patients (Fig. 4d).

### High expression levels of ALCAM, CACNA2D3, DST, EPB41L4A or KIF1B are associated with the favorable prognosis of MYCN non-amplified neuroblastoma patients

**Fig. 5:**
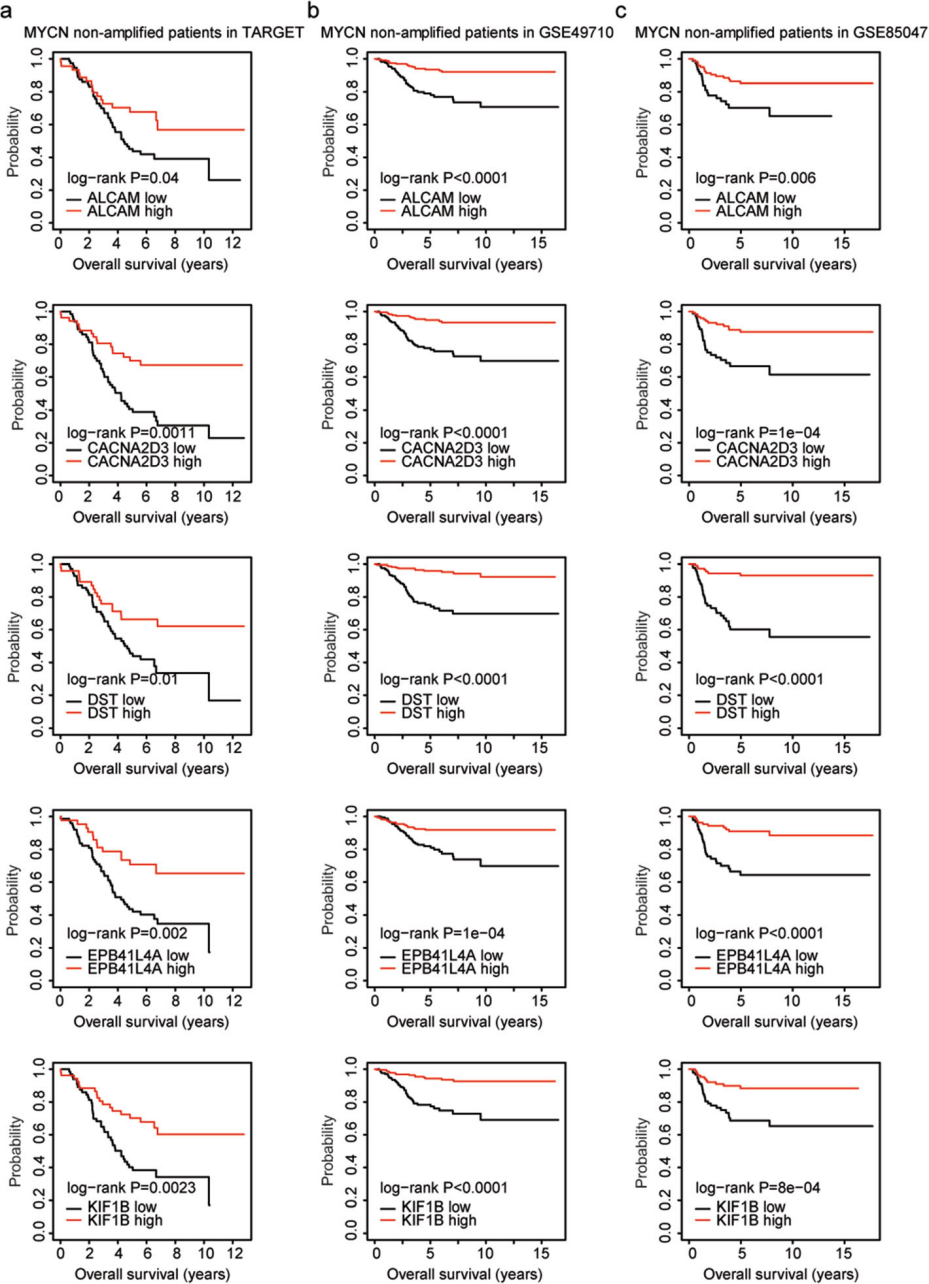
High expression levels of ALCAM, CACNA2D3, DST, EPB41L4A or KIF1B are associated with the favorable prognosis of MYCN non-amplified neuroblastoma patients. **a**-**c** The Kaplan-Meier plots demonstrated the prognostic effects of ALCAM, CACNA2D3, DST, EPB41L4A and KIF1B in MYCN non-amplified pediatric neuroblastoma using TARGET dataset (**a**), GSE49710 dataset (**b**) and GSE85047 dataset (**c**). Patients were divided into two sub-groups based on the mean expression levels of ALCAM, CACNA2D3, DST, EPB41L4A or KIF1B. The log-rank test was used to determine the overall survival P values

Furthermore, we determined the prognostic effects of ALCAM, BTBD9, CACNA2D3, DST, EPB41L4A, FGD6, GMEB1, IGSF3 and KIF1B in MYCN non-amplified neuroblastoma patients. We found that ALCAM, CACNA2D3, DST, EPB41L4A and KIF1B were associated with the favorable prognosis of MYCN non-amplified neuroblastoma patients. MYCN non-amplified neuroblastoma patients with lower expression levels of ALCAM, CACNA2D3, DST, EPB41L4A or KIF1B were with lower overall survival in TARGET dataset (Fig. 5a), GSE49710 dataset (Fig. 5b) and GSE85047 dataset (Fig. 5c).

**Fig. 6:**
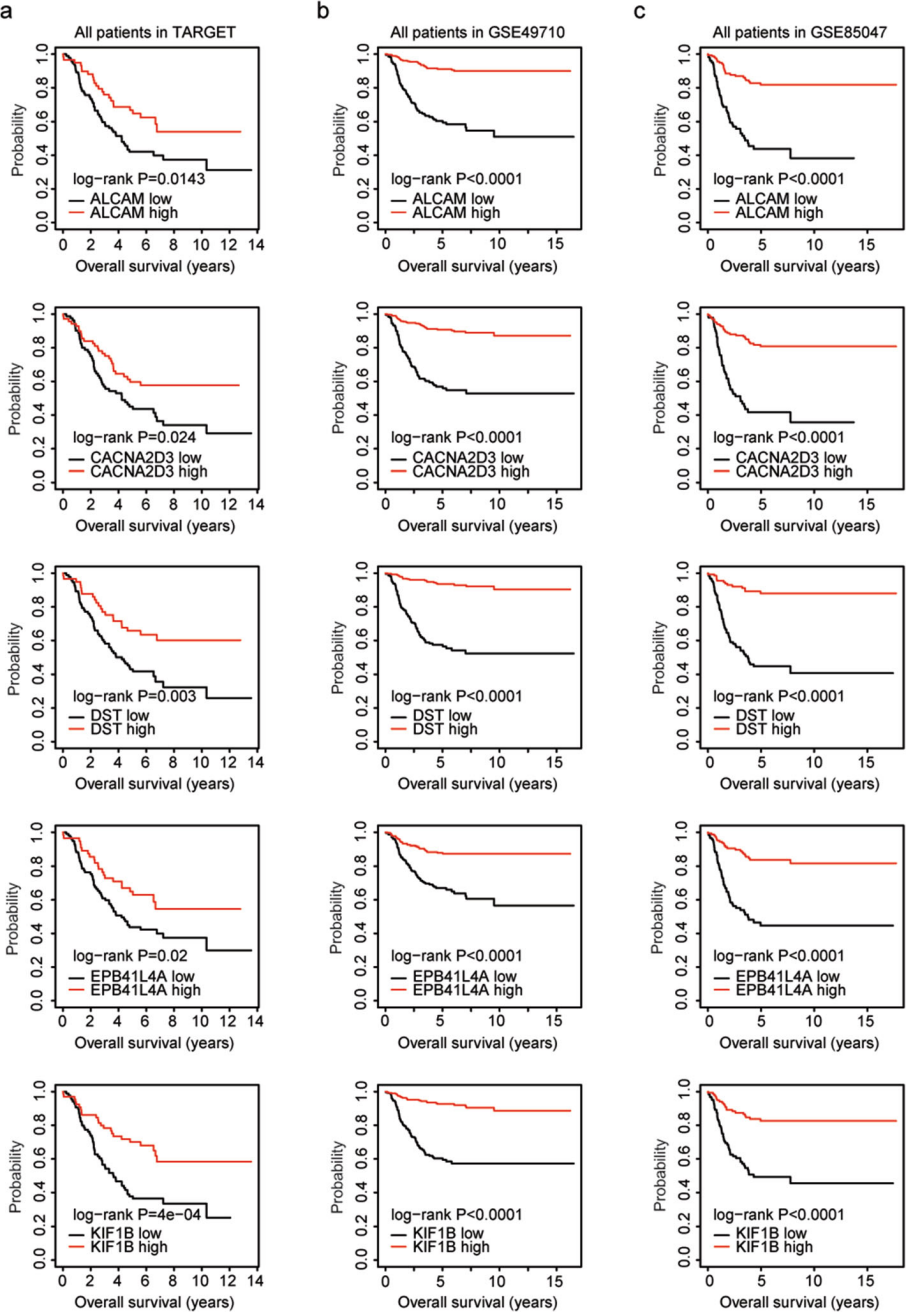
High expression levels of ALCAM, CACNA2D3, DST, EPB41L4A or KIF1B are associated with the favorable prognosis of neuroblastoma patients. The Kaplan-Meier plots demonstrated the prognostic effects of ALCAM, CACNA2D3, DST, EPB41L4A or KIF1B in pediatric neuroblastoma using TARGET dataset (**a**), GSE49710 dataset (**b**) and GSE85047 dataset (**c**)

Interestingly, high expression levels of ALCAM, CACNA2D3, DST, EPB41L4A or KIF1B were not only associated with the prognosis of MYCN non-amplified neuroblastoma patients, but also were associated with the prognosis of all neuroblastoma patients. Neuroblastoma patients with higher expression levels of ALCAM, CACNA2D3, DST, EPB41L4A or KIF1B had better clinical overall survival in TARGET dataset (Fig. 6a), GSE49710 dataset (Fig. 6b) and GSE85047 dataset (Fig. 6c).

### Expression level of DST is an independent prognostic factor in MYCN non-amplified pediatric neuroblastoma

**Fig. 7:**
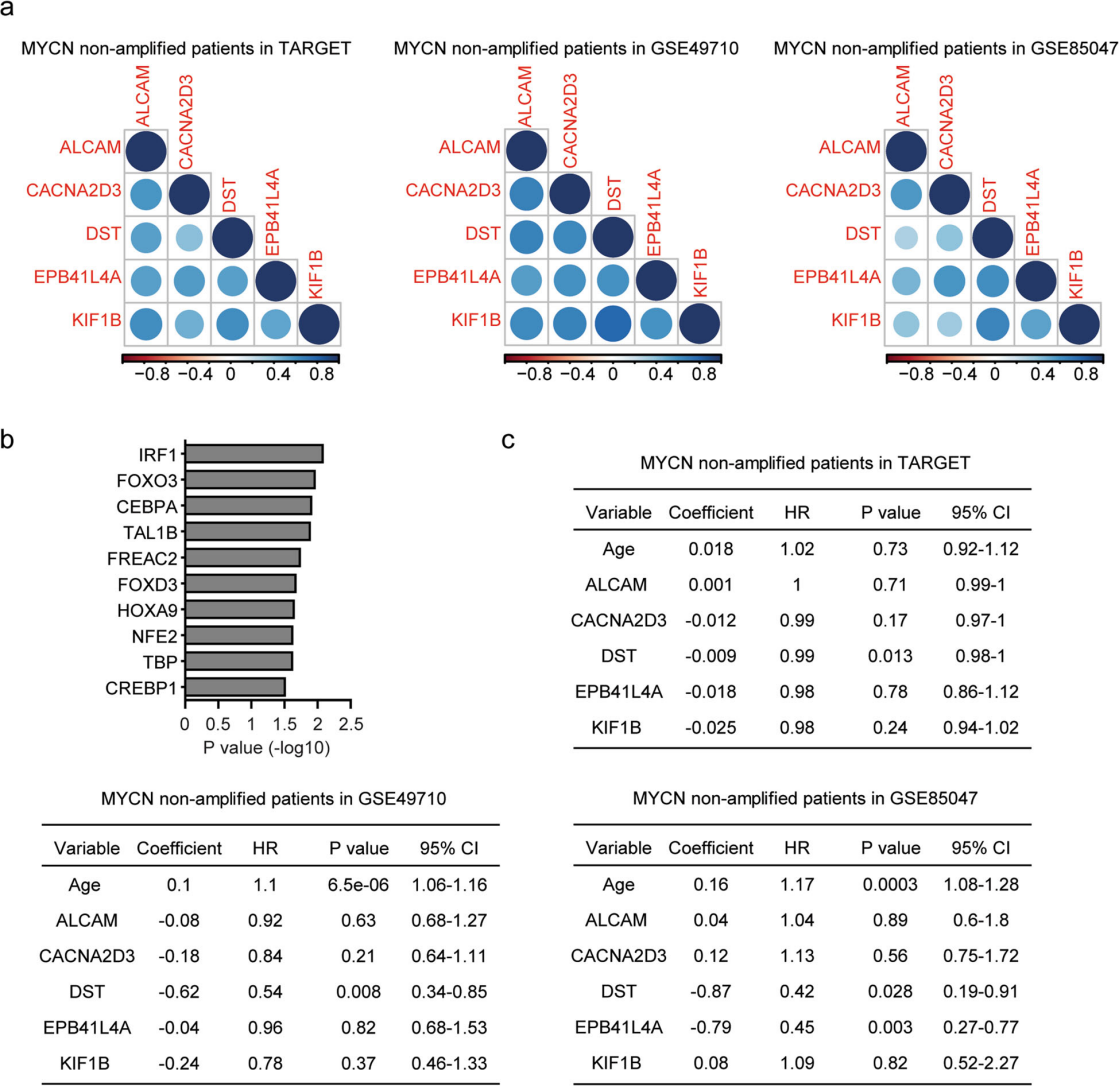
Expression level of DST is an independent prognostic factor in MYCN non-amplified pediatric neuroblastoma. **a** Corrplots demonstrated the associations of ALCAM, CACNA2D3, DST, EPB41L4A and KIF1B in MYCN non-amplified neuroblastoma patients in TARGET, GSE49710 and GSE85047 datasets. The color of the circle represented the correlation coefficients. **b** The enriched transcription factors of ALCAM, CACNA2D3, DST, EPB41L4A and KIF1B genes. **c** Multivariate cox regression was used to determine the correlation of age, ALCAM, CACNA2D3, DST, EPB41L4A or KIF1B expression levels and overall survival in MYCN non-amplified neuroblastoma patients in TARGET, GSE49710 and GSE85047 datasets. HR represented hazard ratio

Next, we tried to determine the associations of ALCAM, CACNA2D3, DST, EPB41L4A and KIF1B in MYCN non-amplified neuroblastoma patients. Based on their expression levels in MYCN non-amplified neuroblastoma patients, we found those genes were highly correlated with each other, as demonstrated the high correlation coefficients of those genes in TARGET, GSE49710 and GSE85047 datasets (Fig. 7a). And ALCAM, DST, CACNA2D3 and KIF1B genes were all predicted to be the target genes of IRF1, FOXO3 or CEBPA transcription factors using DAVID analysis (Fig. 7b).

Furthermore, using multivariate cox regression, we determined the correlations of age, ALCAM, CACNA2D3, DST, EPB41L4A or KIF1B expression in the prediction of the clinical overall survival of MYCN non-amplified neuroblastoma patients. Age alone was an independent prognostic marker of MYCN non-amplified neuroblastoma in GSE49710 and GSE85047 datasets, but not in TARGET dataset (Fig. 7c). In TARGET, GSE49710 and GSE85047 datasets, we found that the expression level of DST was an independent prognostic marker (Fig. 7c). The expression level of EPB41L4A was an independent prognostic marker in GSE85047 dataset (Fig. 7c). Those results suggested that, although, ALCAM, CACNA2D3, DST, EPB41L4A and KIF1B shared similar expression signature, the prognostic significance of DST was independent.

### Younger patients with higher DST expression level have the best prognosis in MYCN non-amplified pediatric neuroblastoma

**Fig. 8:**
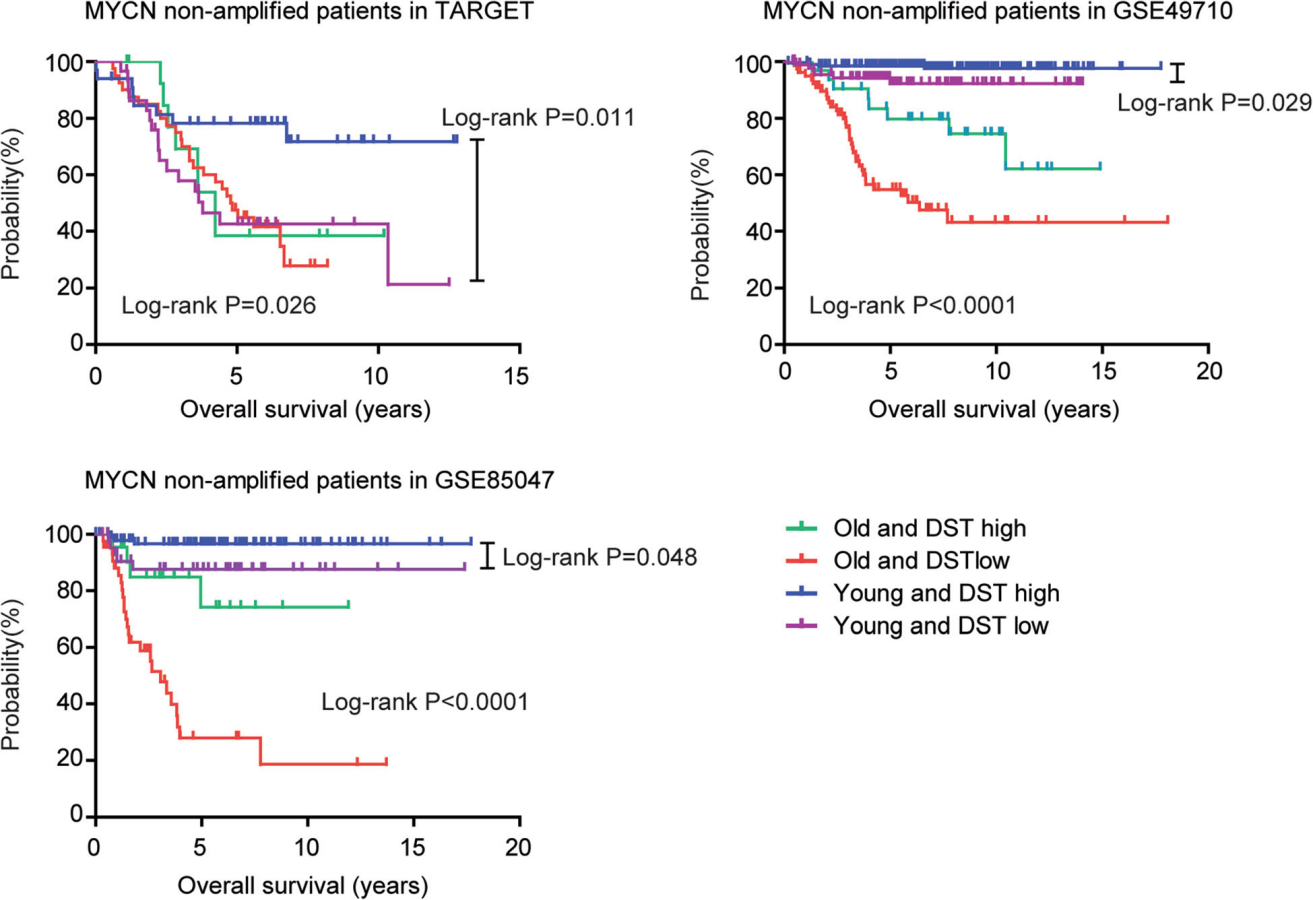
Younger patients with higher DST expression levels have the best prognosis in MYCN non-amplified pediatric neuroblastoma. MYCN non-amplified pediatric neuroblastoma patients in TARGET, GSE49710 and GSE85047 datasets were divided into older patients with higher DST expression, older patients with lower DST expression, younger patients with higher DST expression and younger patients with lower DST expression four sub-groups. The Kaplan-Meier plots determined the different overall survival of those four sub-groups

Since age associated gene DST was an independent prognostic factor in MYCN non-amplified pediatric neuroblastoma, we wondered if the combination of DST, age and MYCN could achieve the best prognostic significance. To test this hypothesis, MYCN non-amplified pediatric neuroblastoma patients in TARGET dataset were divided into older patients with higher DST expression, older patients with lower DST expression, younger patients with higher DST expression and younger patients with lower DST expression four sub-groups. We found that MYCN non-amplified younger patients with higher DST expression had the best prognosis. However, other three sub-groups had similar prognosis in TARGET dataset (Fig. 8). In GSE49710 and GSE85047 datasets, MYCN non-amplified younger patients with higher DST expression also had the best prognosis, while, MYCN non-amplified older patients with lower DST expression had the worst prognosis (Fig. 8).

## Discussion

Using the integrated analysis of TARGET, GSE49710 and GSE85047 datasets, our results showed that MYCN non-amplified pediatric neuroblastoma was heterogeneous, comprised younger and older sub-groups. And MYCN non-amplified younger pediatric neuroblastoma patients had better prognosis. Furthermore, MYCN non-amplified younger patients with higher DST expression levels had the best prognosis. On the contrary, MYCN non-amplified older patients with lower DST expression levels had worse prognosis. These analyses provided deep understanding of the heterogeneity of neuroblastoma.

Dystonin/BPAG1 (DST) is bullous pemphigoid antigen 1, involving the autoimmune response in the development of bullous pemphigoid [25]. DST is also associated with multiple neurological disorders, including Parkinson’s disease [26], Alzheimer’s disease [27], epilepsy, dementia and multiple sclerosis [28], through regulation of cytoskeletal dynamics [29] and cell migration [30]. However, the functions of DST in neuroblastoma are barely known. We found that DST was an independent prognostic factor in MYCN non-amplified pediatric neuroblastoma and highly expressed in MYCN non-amplified younger neuroblastoma patients. Combination of DST, age and MYCN achieved best prognostic effects in neuroblastoma. ALCAM, CACNA2D3, EPB41L4A and KIF1B shared similar expression signature and prognostic effects with DST. Moreover, the prognostic significances of ALCAM, CACNA2D3, EPB41L4A and KIF1B in MYCN non-amplified pediatric neuroblastoma were not previously reported.

The purpose of this study was to determine the prognostic effects of age related genes in neuroblastoma. The present study suggested the relative heterogeneity of MYCN non-amplified pediatric neuroblastoma. And MYCN non-amplified pediatric neuroblastoma could be divided into several sub-groups by age and DST expression levels. However, there were some limitations in this study. The conclusions were generated from published datasets and lack of further validations. Therefore, functions and prognosis of DST in MYCN non-amplified neuroblastoma should be further studied.

## Conclusions

MYCN non-amplified neuroblastoma is a heterogeneous disease and could be divided into sub-groups based on age or the expression levels of ALCAM, CACNA2D3, DST, EPB41L4A and KIF1B. MYCN non-amplified younger neuroblastoma patients with higher DST expression levels have the best clinical overall survival.

## Data Availability

The datasets used and analyzed during the current study are available from the corresponding author on reasonable request and The TARGET datasets were downloaded from https://ocg.cancer.gov/. The GSE49710 and GSE85047 datasets were downloaded from https://www.ncbi.nlm.nih.gov/geo/.

## Abbreviations

TARGET: Therapeutically Applicable Research to Generate Effective Treatments
GEO: Gene Expression Omnibus
TCGA: The Cancer Genome Atlas
DAVID: The Database for Annotation, Visualization and Integrated Discovery
KEGG: Kyoto Encyclopedia of Gens and Genomes
NRC: European Neuroblastoma Research Consortium
DST: Dystonin/BPAG1
HR: hazard ratio
